# Human stromal corneal lenticule implantation for refractory full-thickness macular holes: surgical technique and preliminary outcomes

**DOI:** 10.1038/s41433-026-04729-1

**Published:** 2026-07-11

**Authors:** Rodolfo Mastropasqua, Maria Ludovica Ruggeri, Alberto Quarta, Ruggero Tartaro, Corina Valentina de Sanctis Ciacci, Letizia Pelusi, Mario Nubile

**Affiliations:** 1 Department of Neuroscience, Imaging and Clinical Sciences, University G. D’Annunzio Chieti-Pescara, Chieti, Italy; 2 Eye Clinic, University Hospital of Chieti-Pescara, Chieti, Italy; 3 Department of Medicine and Aging Science Center for Advanced Studies and Technology (CAST), ‘G. d’Annunzio’ University of Chieti-Pescara, Chieti, Italy; 4 Ophthalmology Clinic, Department of Medicine and Aging Sciences, ‘G. d’Annunzio’ University of Chieti-Pescara, Chieti, Italy

**Keywords:** Retinal diseases, Implants

Biological scaffolds have been explored in ophthalmology to improve structural and regenerative outcomes. Refractory full-thickness macular hole (rFTMH) remains a surgical challenge, as currently available techniques primarily aim at anatomical closure without consistently achieving functional recovery [1]. Human stromal corneal lenticules (hSCLs) represent a biocompatible stromal tissue that has previously been used in the treatment of keratoconus, showing promising potential in bioengineering approaches with regenerative factors [2–4]. Earlier studies have described the use of smile-derived hSCLs for the treatment of various vitreoretinal conditions; however, to date, their clinical application remains limited in Europe [5, 6]. Recently, our group described the use of decellularized corneal lenticules in FTMH in an ex vivo porcine FTMH model, demonstrating successful human stromal corneal lenticules (hSCLs) implantation through both clinical and histological analyses [7].

The aim of this study was to describe the surgical technique for hSCL implantation in refractory macular holes. Six eyes from six patients affected by rFTMH and addressed for repeated surgery were enrolled in this prospective interventional pilot study at the University Hospital of Chieti-Pescara, Italy.

## Human stromal corneal lenticule preparation

Stromal lenticules were created using a 500-kHz femtosecond laser platform (VisuMax, Carl Zeiss Meditec, Jena, Germany) from eye-bank corneoscleral tissue not suitable for optical keratoplasty but meeting standard safety criteria. A 100-µm-thick lamellar dissection was performed, after epithelial removal, with corneoscleral rims mounted on an artificial anterior chamber, using standard anterior lamellar keratoplasty settings and the stromal lamella was subsequently isolated and resized to a 1.0-mm circular lenticule using a sterile dermatologic punch. The resulting lenticule consisted of a Bowman layer on one surface and anterior stromal tissue on the opposite side.

## The surgical technique

All surgeries were performed by the same fellowship-trained vitreoretinal surgeon (RM) (Supplemental Video). Under retrobulbar anaesthesia and standard antisepsis, a 25-gauge revision vitrectomy was performed with careful removal of any residual vitreous.

The superotemporal trocar was then enlarged to a 1-mm sclerotomy using an MVR blade to allow the lenticule insertion. The hSCL was introduced into the vitreous cavity using Eckardt ILM forceps. With the infusion line temporarily stopped, the lenticule was positioned beneath the macular hole using ILM forceps, ensuring complete 360° coverage of the defect. During positioning, the lenticule was oriented with the smooth Bowman surface facing upward and the stromal dissected surface toward the retinal pigment epithelium (RPE), to facilitate adhesion and scaffold integration. Correct positioning was confirmed by intraoperative OCT.

The sclerotomy was sutured, followed by fluid–air exchange and tamponade with 20% sulphur hexafluoride (SF₆). Patients were instructed to maintain a face-down position for 3 days.

## Results

**Fig. 1:**
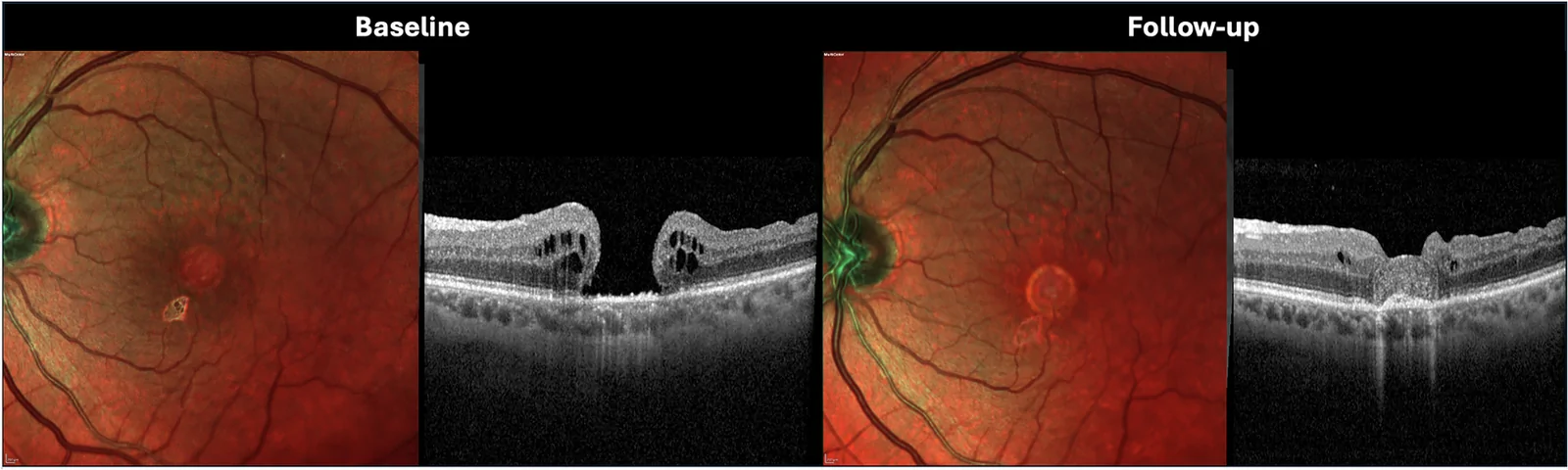
Representative case. Preoperative and postoperative OCT imaging demonstrating successful hSCL positioning and macular hole closure.

**Fig. 2:**
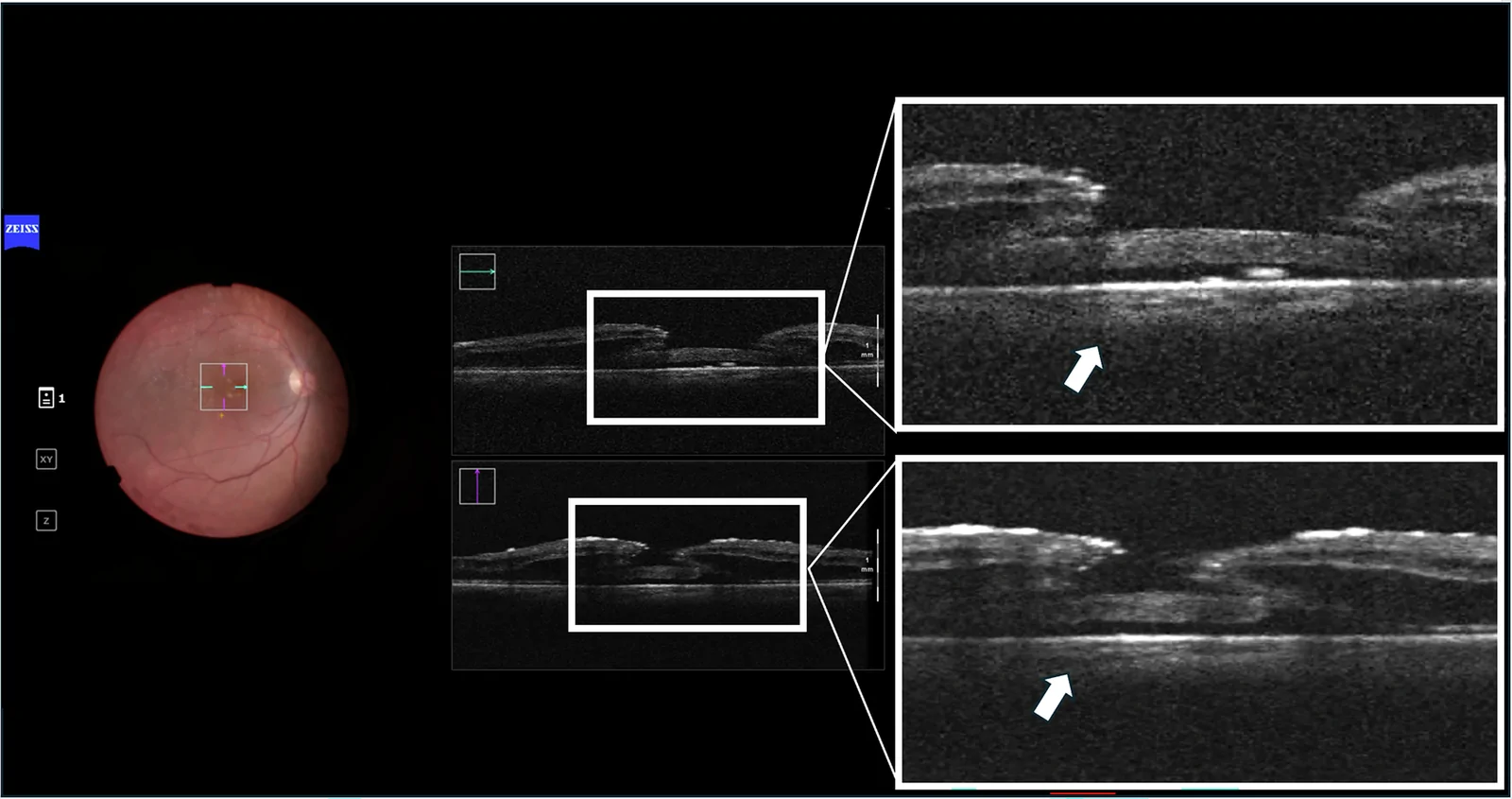
Intraoperative view. Intraoperative OCT confirming correct submacular positioning of the hSCL. The white square indicates a magnified view of the foveal region and the white arrow highlights the lenticule within the macular hole.

Anatomical closure was achieved in all cases at 1-month follow-up. (Fig. 1) Intraoperative and postoperative OCT confirmed stable lenticule integration, consistently showing a hyperreflective subfoveal band and complete 360° coverage beneath the retinal edges. (Fig. 2) No intraoperative or early postoperative complications were observed. These findings suggest early structural integration with stable retinal architecture at short-term follow-up.

These results support the feasibility of hSCL implantation in rFTMH. While previous reports have explored heterogeneous applications of lenticule implantation, this approach focuses on the standardisation of a refined surgical technique specifically for refractory macular holes. Compared with amniotic membrane–based approaches, stromal lenticules may offer a more structurally stable scaffold derived from native ocular stroma, potentially favouring more consistent tissue integration.

This approach introduces a biologically derived stromal scaffold, potentially expanding current surgical strategies beyond purely mechanical closure. Future developments may include the use of stromal lenticules derived from living donors within dedicated tissue banking platforms, enabling standardised and potentially bioengineered applications.

Supplemental material is available at Eye’s website.

## Supplementary information

## Data Availability

All data generated or analysed during this study are included in this article.
