# Supplementary material for: Human stromal corneal lenticule implantation for refractory full-thickness macular holes: surgical technique and preliminary outcomes
Source: Eye (Lond). 2026 Jul 11;40(13):2117–9. doi: 10.1038/s41433-026-04729-1 (PMC13538633; doi:10.1038/s41433-026-04729-1)
Supplement: Supplementary file 1 [file 41433_2026_4729_MOESM2_ESM.docx]

**Supplemental Video. Surgical technique.**

The video reports the step-by- step surgical technique for human stromal corneal lenticule implantation for refractory full thickness macular holes.
